# Neuroprotection by Acetyl-11-Keto-β-Boswellic Acid, in Ischemic Brain Injury Involves the Nrf2/HO-1 defense Pathway

**DOI:** 10.1038/srep07002

**Published:** 2014-11-11

**Authors:** Yi Ding, MinChun Chen, Min Wang, MingMing Wang, Tiejun Zhang, Jongsun Park, YanRong Zhu, Chao Guo, YanYan Jia, YuWen Li, AiDong Wen

**Affiliations:** 1 Department of Pharmacy, Xijing Hospital, Fourth Military Medical University; 2 Department of Pharmacology, Chungnam National University; 3 Department of Pharmacology, School of Pharmacy, Fourth Military Medical University

## Abstract

Stroke is a complex disease involved oxidative stress-related pathways in its pathogenesis. The nuclear factor erythroid-2-related factor 2 (Nrf2)/heme oxygenase-1 (HO-1) pathway has been considered a potential target for neuroprotection in stroke. Acetyl-11-Keto-β-Boswellic Acid (AKBA) is an active triterpenoid compound from the extract of *Boswellia serrate*. The present study was to determine whether AKBA, a novel Nrf2 activator, can protect against cerebral ischemic injury. The stroke model was produced in Sprague–Dawley rats via middle cerebral artery occlusion. To model ischemia-like conditions *in vitro*, primary cultured cortical neurons were exposed to transient oxygen and glucose deprivation (OGD). Treatment of AKBA significantly reduced infarct volumes and apoptotic cells, and also increased neurologic scores by elevating the Nrf2 and HO-1 expression in brain tissues in middle cerebral artery occlusion (MCAO) rats at 48 hours post reperfusion. In primary cultured neurons, AKBA increased the Nrf2 and HO-1 expression, which provided protection against OGD-induced oxidative insult. Additionally, AKBA treatment increased Nrf2 binding activity to antioxidant-response elements (ARE). The protective effect of AKBA was attenuated by knockdown of Nrf2 or HO-1. In conclusion, these findings provide evidence that AKBA protects neurons against ischemic injury, and this neuroprotective effect involves the Nrf2/HO-1 pathway.

Ischemic stroke due to occlusion of brain vasculature is a leading source of mortality amounting to 9% of total deaths each year^1^. Although thrombolysis is the sole presently effective available stroke treatment, it is limited to a small proportion of patients with stroke because it carries the risk of intracranial hemorrhagic transformation. The importance of developing an effective treatment remains essential. Evidence has accumulated that excessive reactive oxygen species (ROS) are closely related to cerebral ischemia/reperfusion (I/R) injury in stroke^2^. Brain tissue is particularly susceptible to oxidative damage. Thus, antioxidants are considered in treatment and prevention of stroke.

Acetyl 11-keto-β-boswellic acid (AKBA), a pentacyclic triterpenoid compound, is among the most important active principles within the multi-component mixture of *Boswellia serrata* resin^3^. *Boswellia serrata* resin extracts (Boswellic acids) shows an *in vivo* antioxidant activity in many conditions that include bowel disease^4^, myocardial I/R injury^5^ and pulmonary fibrosis^6^. The neuroprotective property of pentacyclic triterpenoid has attracted increasing attention recently. For example, oleanolic acid shows protective effects on cerebral ischemic damage and H_2_O_2_-induced injury *in vitro*^7^. Recently, it was reported that ursolic acid, a naturally occurring pentacyclic triterpenoid, promotes the neuroprotection after cerebral ischemia in mice by activating Nrf2 pathway^8^. Another study revealed that AKBA may have a better antioxidant effect compared with ursolic acid in mice^9^. Based on these studies, we hypothesized that AKBA, which has the similar chemical structure to ursolic acid, may promote the neuroprotection via the Nrf2 pathway.

Nrf2 controls the coordinated expression of important antioxidant and detoxification genes through a promoter sequence termed the antioxidant response element (ARE). Phase II genes, including heme oxygenase-1 (HO-1), glutathione S-transferases (GSTs) and NAD(P)H quinone oxidoreductase, work in synergy to provide protection by regulating and maintaining intracellular redox states^10^,^11^. Of these, HO-1 has been reported to have the most AREs on its promoter, making it a highly effective therapeutic target for protection against neurodegenerative diseases. It gives protection in part by degrading its pro-oxidant substrate, heme, and generating the antioxidants biliverdin and bilirubin^12^. Recently, studies have provided evidence for the therapeutic potential of targeting the Nrf2/HO-1 pathway in brain injury after ischemic stroke^13^,^14^.

Considering the beneficial properties of triterpenoids as well as the possible role of the Nrf2/HO-1 pathway, in this study we applied *in vivo* and *in vitro* ischemic paradigms to analyze the protective properties of AKBA. We hypothesized that AKBA would provide neuroprotection against I/R injury induced by transient middle cerebral artery occlusion (MCAO) in rats and oxygen and glucose deprivation (OGD) in primary cultured neurons, and that the protection would occur through activation of the Nrf2/HO1 pathway.

## Methods

### MCAO model

All procedures involving animals were approved by the Institutional Animal Care and Use Committee of the Fourth Military Medical University and written up following the ARRIVE guidelines. Experiments were performed in accordance with published National Institutes of Health guidelines. Adult male Sprague-Dawley rats aged 8–10 weeks from the Experimental Animal Center of Fourth Military Medical University (Xi'an, China) (280–300 g). All rats were divided randomly into the following 3 groups using a random number table generated by SPSS 16.0 (SPSS Inc., Chicago, IL, USA): sham-operated group (sham), vehicle-treated I/R group (vehicle + I/R), and AKBA-treated I/R group (AKBA + I/R). In all the three groups, eight rats were used for physiologic parameters and infarct size measurement, eight rats were used for HE staining and TUNEL staining, eight rats were used for determination of oxidative stress, six rats were used for Western blotting, and six rats were used for immunostaining.

Rats were anesthetized using 2.0 to 3.0% isoflurane and maintained using 1.0 to 1.5% isoflurane (both in 70% N_2_O/30% O_2_). Focal cerebral ischemia was performed using the method of right MCAO with an intraluminal filament as described previously^15^. Cerebral blood flow (CBF) was monitored using laser Doppler flowmetry (Perimed AB, PeriFlux System 5000, Stockholm, Sweden) in the ipsilateral cortex (2 mm posterior and 5 mm lateral to bregma). Sham operated rats were manipulated in the same way, but the MCA was not occluded. Animals that did not show a CBF reduction of at least 70% and animals that died after ischemia induction were excluded from the groups. At 2 h after the induction of ischemia, the filament was slowly withdrawn. The neck incision was closed and rats were allowed to recover. After revival from anesthesia, the animals were put back into cages with the room temperature maintained at 25 ± 2°C. The animals were allowed to survive for 2 days with free access to water and food. Mean arterial blood pressure, pH, arterial blood gases, and blood glucose levels during treatment were evaluated.

**Figure 1:**
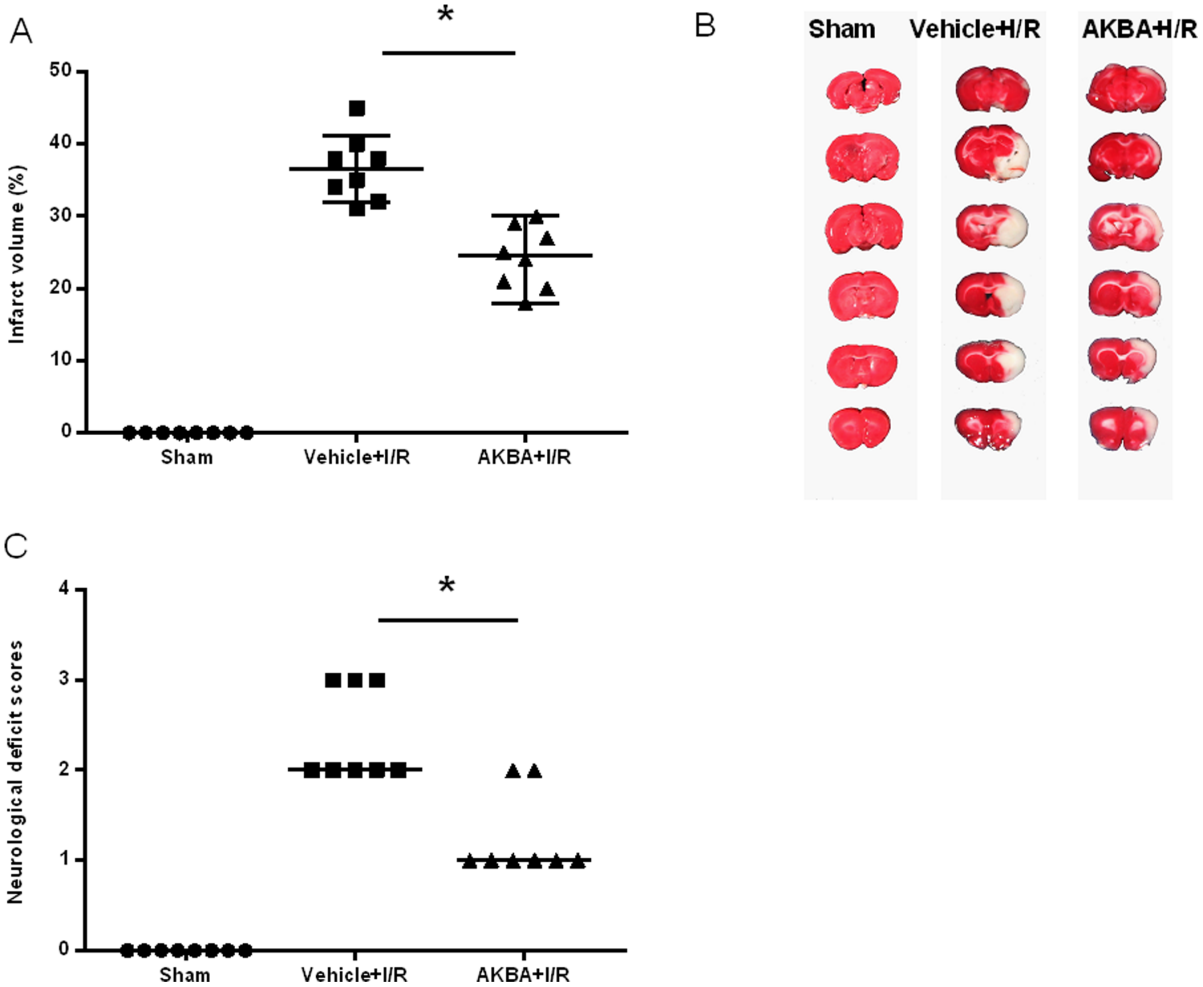
AKBA protects against cerebral ischemia reperfusion injury in MCAO rats. (A) Statistical analysis of the percentage of infarct volume was determined for each study group (data represent the mean ± SD). (B) Representative 2,3,5-triphenyltetrazolium chloride (TTC) staining of the cerebral infarct in the rat brain. (C) Scatterplot of neurological deficit scores at 48 hours after reperfusion. Median of each data series is represented by a horizontal line. Data were analyzed using a nonparametric method (Kruskal–Wallis test). (n = 8 animals for each group). *, P < 0.05 vs vehicle + I/R.

AKBA (reagent grade, purity > 90%, Santa Ana, CA) diluted with physiological saline (20 mg/kg) was administered by intraperitoneal injection. Vehicle of 2 ml/kg physiological saline (vehicle + I/R group) and 20 mg/kg AKBA (AKBA + I/R group) were given immediately after the onset of reperfusion. The dose of 20 mg/kg AKBA administered to rats (corresponding to about 350 mg *Boswellia serrata* extract/kg) was chosen based on previous study^4^. Meanwhile, in a preliminary experiment, a dose-response (5 mg/kg, 10 mg/kg and 20 mg/kg administered by intraperitoneal injection) study was conducted (data was shown in Figs. 1). From infarct volume measurement, we demonstrated that the dosage of AKBA at 20 mg/kg the best therapeutic effects among three doses, and therefore we focused on the AKBA treatment at 20 mg/kg for biochemical and molecular analysis.

### Neurological function evaluation and quantification of infarct volume

According to the Zea Longa standard^15^, neurological deficits were blindly evaluated 48 h after reperfusion with a 5-point scale system: 0, no deficit; 1, not being able to completely stretch the contralateral torso and forelimb; 2, turning to the ipsilateral side when held by the tail; 3, falling over to the affected side; 4, not being able to walk and no spontaneous locomotor activity.

To calculate infarct volume, brains were removed at 48 h after MCAO and were cut into 2 mm thick coronal sections and subjected to 2, 3, 5-triphenyltetrazolium chloride (TTC) staining. Unstained areas were defined as infarcts and were measured using image analysis software (Adobe Photoshop CS3, San Jose, CA, USA). The percentage of the infarct volume was calculated by the following formula: ([total contralateral hemispheric volume]- [total ipsilateral hemispheric stained volume])/(total contralateral hemispheric volume) × 100%.

### HE and TUNEL staining

Hematoxylin and eosin (HE) staining was performed to reveal the morphological features of injured neurons in cerebral cortex. TUNEL staining was performed on paraffin-embedded sections. Commercially available reagents (Promega, DeadEnd Flurometric Tunel System) were used to perform TUNEL analysis. The total number of TUNEL positive neurons in the ipsilateral hemisphere was counted in three different fields for each section by an investigator who was blinded to the studies by light microscopy.

### Determination of oxidative stress

Animals were killed at 48 h after reperfusion and brains were removed rapidly. Right cortical samples (n = 8 for each group) were weighed. Homogenates were centrifuged at 15,000 g for 10 minutes, and the supernatant obtained was used for the following measurements. The superoxide dismutase (SOD) activity was determined spectrophotometrically using commercially available assay kits obtained from Cayman Chemicals (Ann Arbor, MI, USA). Malondialdehyde (MDA) were measured in deproteinized brain extracts by high performance liquid chromatography (HPLC)^16^. The chromatography system consisted of Agilent 1200 Series LC System equipped with a UV detector, an autosampler (Agilent Technologies, USA). Chromatographic separation was achieved on a Dikma column (Dikma, Beijing, China. 150 mm × 4.6 mm, 5 μm).

### Immunofluorescence staining

The paraffin sections, 5 mm thick, which were drawn 48 h after reperfusion, were first deparaffinized in xylene, rehydrated with various grades of ethanol, and pretreated with 10 μg/ml proteinase K for 30 min at 37°C. By incubating the sections in 10% bovine serum albumin, nonspecific binding of immunoglobulins was blocked for 20 min. Then the sections were incubated overnight at room temperature in with anti-Nrf2 (ab31163, Abcam) or anti-HO-1 (ab13243, Abcam), and with anti-NeuN antibody to mark to neurons (monoclonal clone A60, Chemicon). Sections were then incubated with Alexa Fluor s 488 anti-rabbit IgG or Alexa Fluor s 594 anti-mouse IgG secondary antibodies for 2 h. Then, the sections were incubated with DAPI for nuclear counterstaining. Finally, the sections were coverslipped. The stained sections were examined under a fluorescence microscope (Olympus, Tokyo, Japan).

### Western blot analysis

Right cortical samples were weighed and protein was extracted. Cortical neurons were washed twice with ice-cold PBS. Nuclear protein of Nrf2 was obtained using a Neuclear-Cytosol Extraction Kit (Applygen Technologies Inc., Beijing) and total protein of HO-1 in extraction was obtained using Total Protein Extraction Kit (Applygen Technologies Inc., Beijing) following the manufacturer's protocols. Equal amounts of total protein extracts or nuclear protein extracts were separated by SDS-PAGE and transferred onto polyvinylidenedifluoride membranes (Millipore, Bedford, MA, USA) by electrophoresis, and membranes were blocked with 5% nonfat milk in TBST (0.1% Tween 20 in TBS) for 1 h at room temperature. Blots were then incubated with the antibody for Nrf2, HO-1, Histone H3 (Cell Signaling Technology) and β-actin (Cell Signaling Technology).

### Primary culture of rat cortical neurons

Cortical neurons were prepared from brains of one-day-old Sprague-Dawley rats. Approximately 30,000 cells in 50 ml neurobasal medium containing glutamine (1 mmol/L), 1% penicillin, streptomycin (Pen/Strep), and 10% fetal bovine serum were seeded into 6-well plates. After 2 h, 0.5 ml neurobasal medium containing the serum-free B27 supplement (2%), Pen/Strep, and glutamine were added to each well. After 2 days *in vitro*, 5 mM cytosine arabinofuranoside was added to inhibit glial proliferation. At 5 days *in vitro*, the medium was changed to fresh neurobasal medium containing B27. Neurons were cultured at 37°C in a humidified 5% CO_2_ atmosphere and used after 7 days *in vitro*.

### Oxygen-glucose deprivation and reoxygenation

Cells seeded in 96-well plates were pretreated with AKBA at the indicated concentrations for 2 h. Then, to model ischemia-like conditions *in vitro*, primary cultured cortical neurons were exposed to transient OGD for 60 min. Finally, the neurons were incubated again in the incubator with 95% air and 5% CO_2_ with or without AKBA for an additional 24 h. OGD-induced cell death was quantified using the 3-[4,5-dimethylthiazol-2-yl]-2,5-diphenyltetrazolium bro-mide (MTT) assay. Intracellular ROS levels were measured using the dye 2′,7′-dichlorofluorescein diacetate (DCF-DA) as described previously^17^.

### Nrf2 and HO-1 siRNA transfection

Neuronal cells were transiently transfected with siRNA targeting to Nrf2 or HO-1 by Lipofectamine 2000 TM (Invitrogen) according to the manufacturer's protocol. Knockdown efficiency of Nrf2 and HO-1 was determined by Western blot analysis. Cells were transfected with siRNA or with a nontargeting scramble control siRNA for 48 h, followed by treatment with AKBA for the indicated times. Rat Nrf2-specific siRNA (5′-ACGCAGGAGAGGGAAGAAUAAAGUU-3′ and 5′-AACUUUAUUCUUCCCUCUCCUGCGU-3′), rat HO-1-specific siRNA (5′-AUGGCAUAAAUUCCCACUGCCACGG-3′ and 5′-CCGUGGCAGUGGGAAUUUAUGCCAU-3′) and a nonspecific siRNA (5′-AUGCACGAUAUAACCUCACCGUCGG-3′ and 5′-CCGACGGUGAGGUUAUAUCGUGCAU-3′) were provided by Invitrogen (Carlsbad, CA, USA). Cell samples were then collected for Western blot analysis, MTT assay and measurement of intracellular ROS levels.

### Electrophoretic mobility shift assay (EMSA)

EMSA was performed using the commercial Chemiluminescent EMSA kit (Pierce Biotechnology). For EMSA, 5 mg of total extracted nuclear proteins was incubated with 1 pmol double stranded ATP end-labeled oligonucleotide probe containing the sequences in binding buffer (10 mM HEPES, pH 7.9, 80 mM NaCl, 3 mM MgCl_2_, 0.1 mM EDTA, 1 mM dithiothreitol, 1 mM phenylmethylsulfonyl fluoride, and 10% glycerol). After the incubation, the samples were loaded on a 5% TBE-polyacrylamide gel (Bio-Rad) and electrophoretically separated in 0.5× TBE buffer. Levels of Nrf2 DNA binding activity were quantified by computer-assisted densitometric analysis.

## Statistical Analysis

The statistical analyses were performed using SPSS 16.0 (SPSS Inc., Chicago, IL, USA). All of the values were presented as mean ± standard deviation (SD), except for the neurological deficit score, and differences between groups were compared with one-way ANOVA followed by followed by Fisher's post hoc test. P < 0.05 was regarded statistically significant. Neurological deficit scores were expressed as the median (range) and were analyzed using a nonparametric method (Kruskal–Wallis test) followed by the Mann–Whitney U-test using Bonferroni correction.

## Results

### AKBA protects against cerebral ischemic injury

AKBA (20 mg/kg) was injected intraperitoneally to rats 2 h after ischemia to evaluate the in vivo neuroprotective effect. The infarct volume of the ipsilateral brain was measured 48 h later with TTC staining. Between vehicle + IR group and AKBA + IR groups, no significant differences in physiological variables (Table 1). AKBA significantly decreased infarct volumes 48 h after reperfusion (Fig. 1A and B). As shown in Figure 1B, AKBA treatment could statistically reduce the infarct volume from 36.6 ± 4.6% (AKBA + IR group) to 24.3 ± 4.3% (vehicle + IR group) (P < 0.05; n = 8 animals per group). In terms of evaluation of neurological function, neurological deficit grading system was carried out. From Fig. 1, rats from the sham group did not have any neurological deficit, and therefore the animals had a neurological score of zero. For the rats in the vehicle + IR group, they remained with the highest neurological deficit score. In agreement with infarct volume measurement, AKBA treatment significantly reduced the neurological deficit score compared with the vehicle treatment (Fig. 1C) (n = 8 animals per group; P < 0.05).

**Figure 2:**
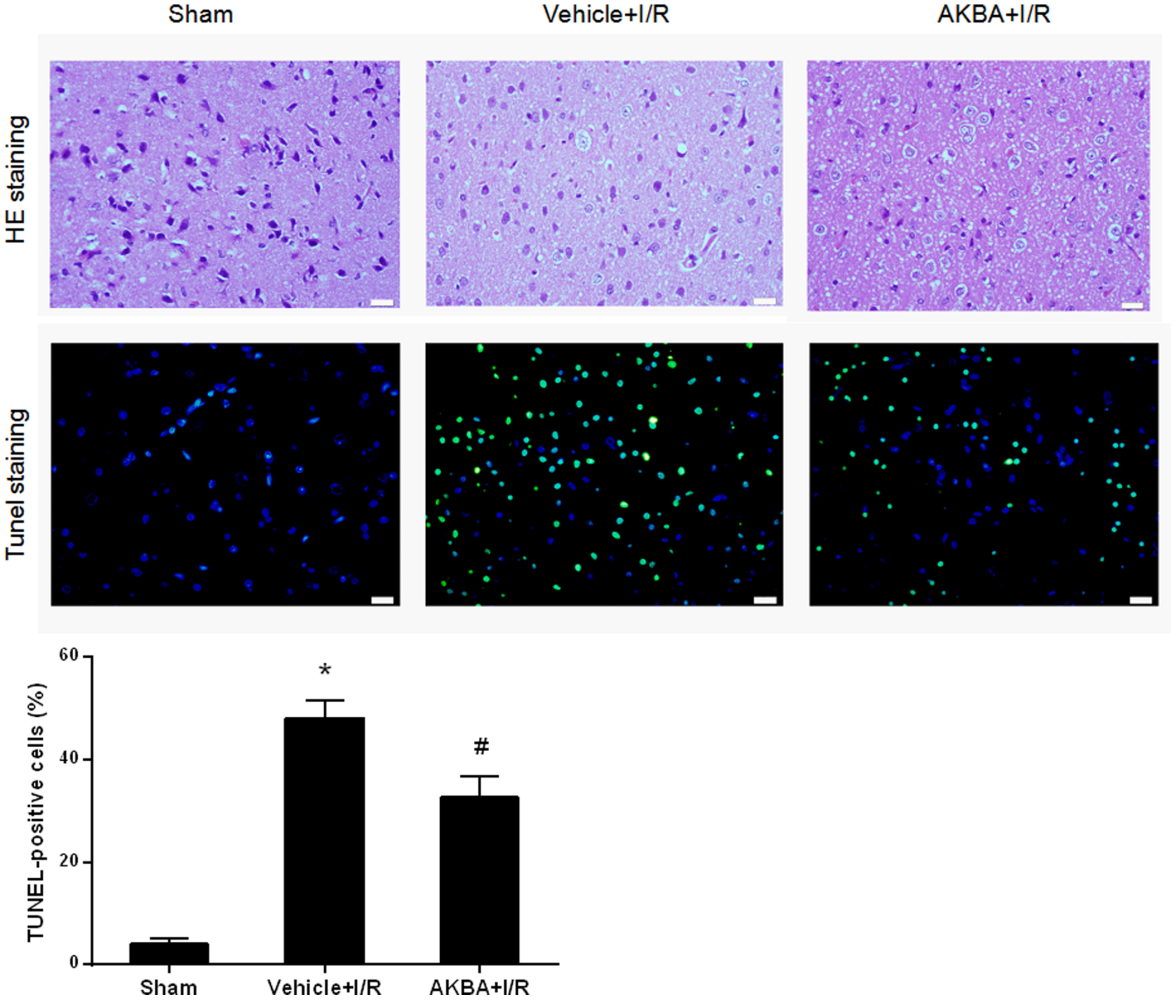
Representative images of HE staining and TUNEL staining performed on sections from ischemic cortex at 48 h after ischemia/reperfusion in rats (TUNEL-positive cells in green, DAPI in blue; Scale bar = 20 μm). Quantitative analysis of TUNEL-positive cells are also exhibited. (n = 8 animals for each group). *, P < 0.05 vs sham and #, P < 0.05 vs vehicle + I/R.

Furthermore, the protective effect of AKBA against cerebral ischemic damage was confirmed by TUNEL staining and HE staining on sections from ischemic cortex at 48 h after ischemia/reperfusion in rats (Fig. 2). Representative photomicrographs of HE staining are shown in Fig. 2. The cells of cortex in sham rats showed an orderly arrangement. Moreover, the cell outline was clear and the structure was compact, and the nucleolus was clearly visible. In vehicle group, the number of cells was decreased and the cells were arranged irregularly in ischemic peri-infarct of cerebral cortex. Most of them were shrunken with a triangulated pycnotic nucleus. In contrast, neuronal damage was substantially reduced in the AKBA + I/R group.

To detect DNA fragmentation in situ, we performed TUNEL staining in brain cryosections. AKBA reduced ischemia-induced DNA damage after 48 h of reperfusion. In control group, TUNEL-positive cells were densely distributed in the ischemic cortex. The AKBA-treated group exhibited a smaller amount of TUNEL-positive cells. The percentage of TUNEL-positive cells in the ischemic cortex was decreased from 48.1 ± 3.3% to 32.6 ± 4.1% by AKBA treatment (n = 8 animals per group; P < 0.05). The percentage was calculated as follows: (number of apoptotic cells/total number counted) × 100%.

### AKBA attenuated oxidative stress

**Figure 3:**
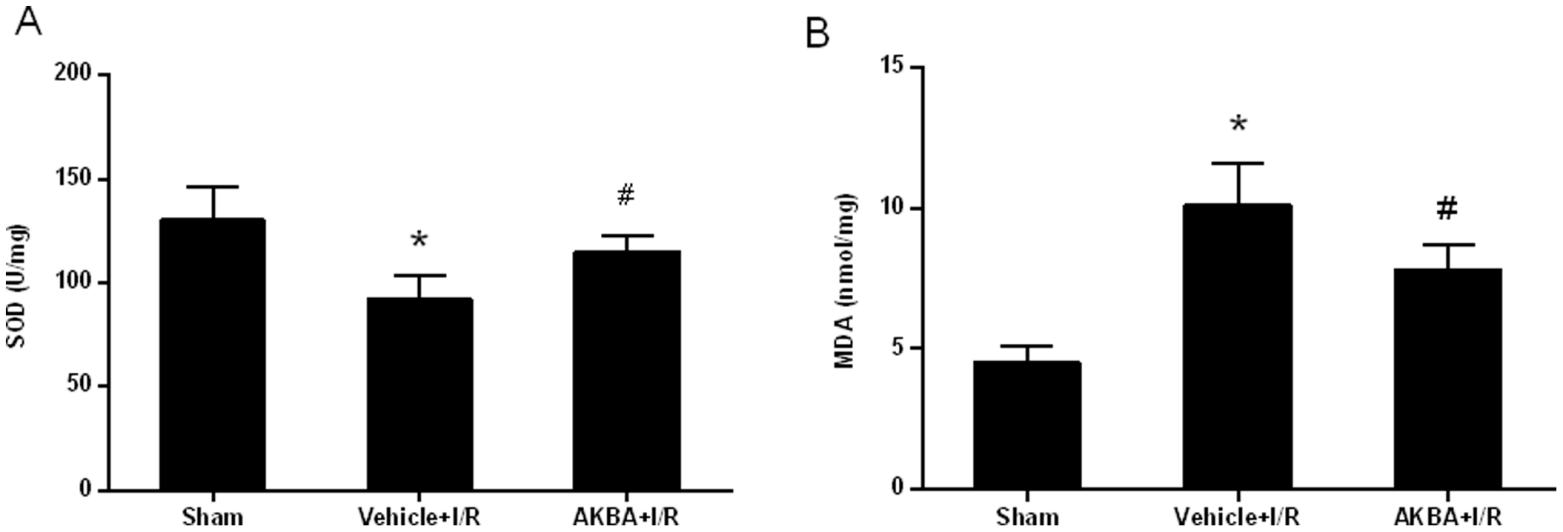
Effect of AKBA on oxidative stress. (A), Assay of SOD content in the cortex at 48 h after reperfusion. (B), Assay of MDA activity in the cortex at 48 h after reperfusion. Bars represent mean ± SD (n = 8 animals for each group). *, P < 0.05 vs sham and #, P < 0.05 vs vehicle + I/R.

SOD activity in the cortex was decreased in the vehicle group compared with the sham group, which was restored by AKBA (n = 8 animals per group; P < 0.05) (Fig. 3A). The MDA level in the cortex, which is an index of lipid peroxidation, was significantly increased in vehicle group compared with the sham-operated group. An evident reduction of the MDA level was observed in AKBA + I/R group compared with vehicle + I/R group (n = 8 animals per group; P < 0.05) (Fig. 3B).

### AKBA promoted the expression of Nrf2 and HO-1

**Figure 4:**
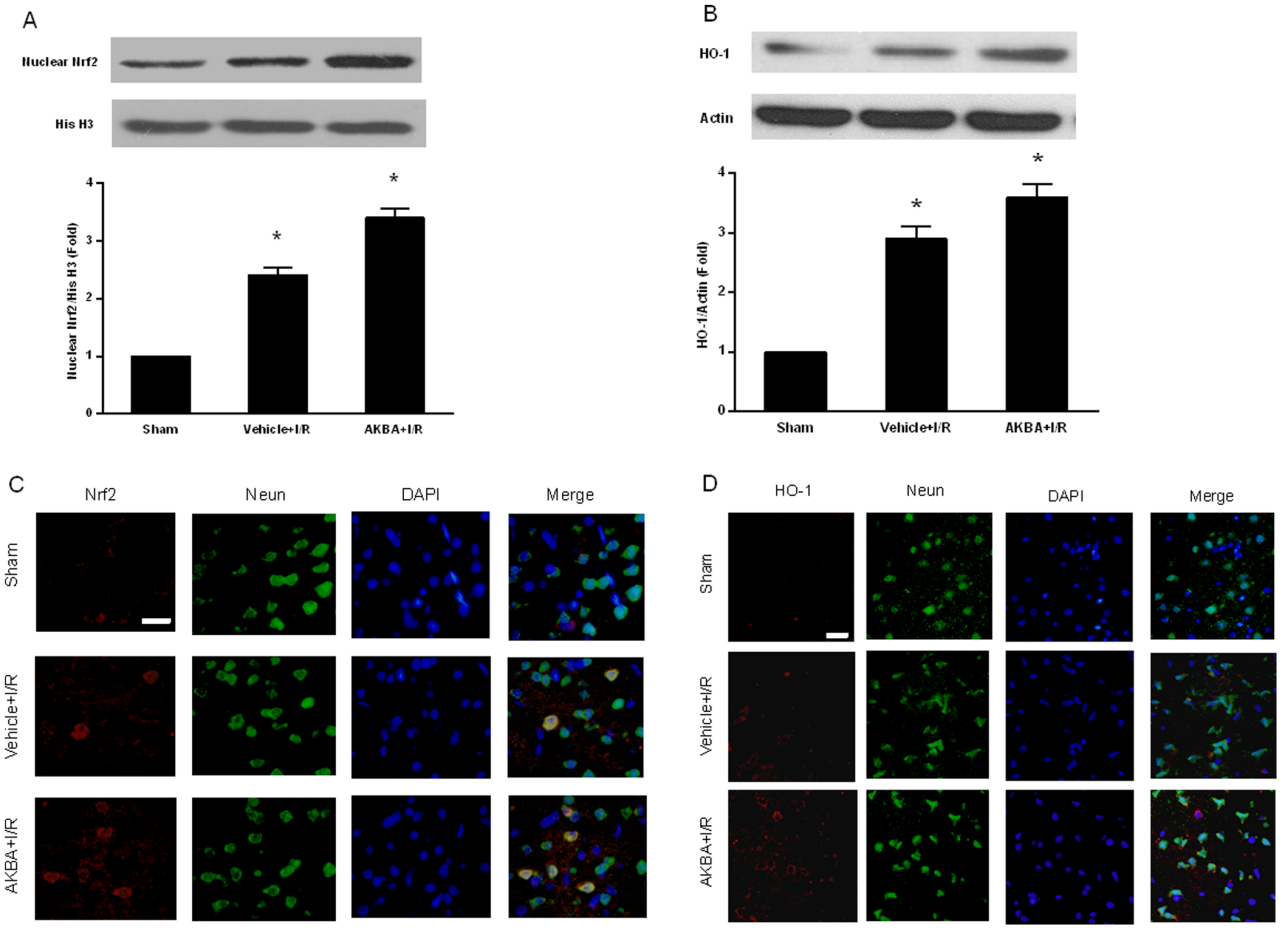
Effect of AKBA on expression of Nrf2 and HO-1 (A) Nrf2 and (B) HO-1 were induced by 50 μM AKBA in the cortex of MCAO rats. Brain cortex tissues were collected at 48 h after cerebral ischemia/reperfusion injury and brain homogenates were evaluated by Western blot for Nrf2, HO-1 and actin. (n = 6 animals per group). *, P < 0.05 vs sham and #, P < 0.05 vs vehicle + I/R. Representative double immunofluorescent stainings for Nrf2/NeuN (C) and HO-1/NeuN (D) are shown in the ischemic cortex at 48 h after reperfusion and in the corresponding regions of sham controls. DAPI-nuclear stainings and merged images are also exhibited. Scale bar = 20 μm.

To identify whether Nrf2/HO-1 signaling is involved in the neuroprotective effect of AKBA, we analyzed ischemic brain tissue by Western blot and immunofluorescence staining. Western blot analysis of cortical tissues at 48 h after MCAO showed that AKBA increased protein expression of nuclear Nrf2 and total HO-1 (n = 6 animals per group; P < 0.05) (Fig. 4A and B).

Consistently, immunofluorescence staining also showed that the expression of Nrf2 and HO-1 in the cortex was upregulated by AKBA at 48 h after ischemia (Fig. 4C and D). In sham group, few cells were stained by Nrf2 and HO-1. In the vehicle + I/R group, the number of cells stained by Nrf2 and HO-1 increased in the ischemic cortex. The number of cells labeled with Nrf2 and HO-1 in the AKBA + I/R group was significantly increased compared with vehicle + I/R group, which indicated that the Nrf2/HO-1 pathway may have a critical role in the AKBA-mediated neuroprotection against I/R injuries in rats.

### AKBA induces HO-1 expression through Nrf2

**Figure 5:**
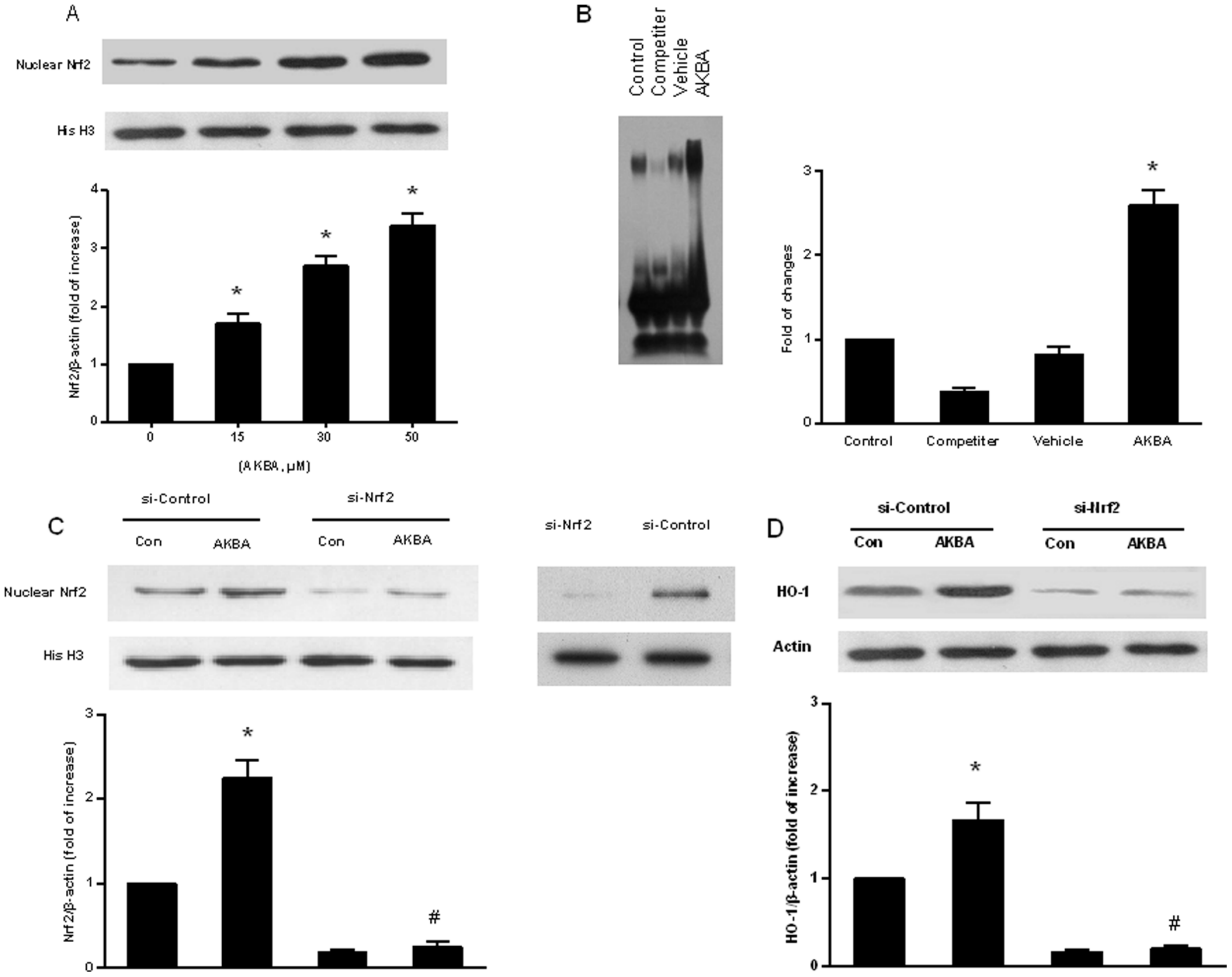
AKBA induces expression of Nrf2 and Nrf2-binding activity in primary cultured neurons. All data represent the mean ± SD of triplicate independent experiments. (A) AKBA induced Nrf2 expression in a concentration-dependent manner. *, P < 0.05 vs control (B) After 2 hour treatment with vehicle or 50 μM AKBA, nuclear extracts were prepared and were used to analyze Nrf2 bingding activity by EMSA. (C) Cells were transiently transfected with control or Nrf2 siRNA for 48 h (transfection efficiency was checked by Western analysis), followed by treatment with 50 μM of AKBA for an additional 8 h. Nuclear extracts were analyzed for Nrf2 levels. (D) Representative immunoblots for HO-1 following 50 μM of AKBA treatment for 24 h in control and Nrf2 siRNA-treated cells. *, P < 0.05 vs si-control group without AKBA and #, P < 0.05 vs si-control group with AKBA.

Nrf2 is an important transcription factor that functions in HO-1 induction. We investigated whether AKBA induced HO-1 expression by activating Nrf2. AKBA increased Nrf2 expression in a concentration dependent manner (Fig. 5A). We next examined the Nrf2 binding activity by EMSA. As expected, treatment of cells with AKBA enhanced Nrf2 binding activity in primary cortical neurons under OGD. Addition of 50 fold molar excess of an unlabeled probe was used as competitor to specifically inhibit the Nrf2 binding (Fig. 5B). The efficiency of the Nrf2 siRNA in knocking down Nrf2 was measured by Western blot (Fig. 5C). Knockdown of Nrf2 decreased Nrf2 and HO-1 expression under either vehicle or AKBA treatment (Fig. 5C and D).

### AKBA protects neurons against Injury Induced by OGD

**Figure 6:**
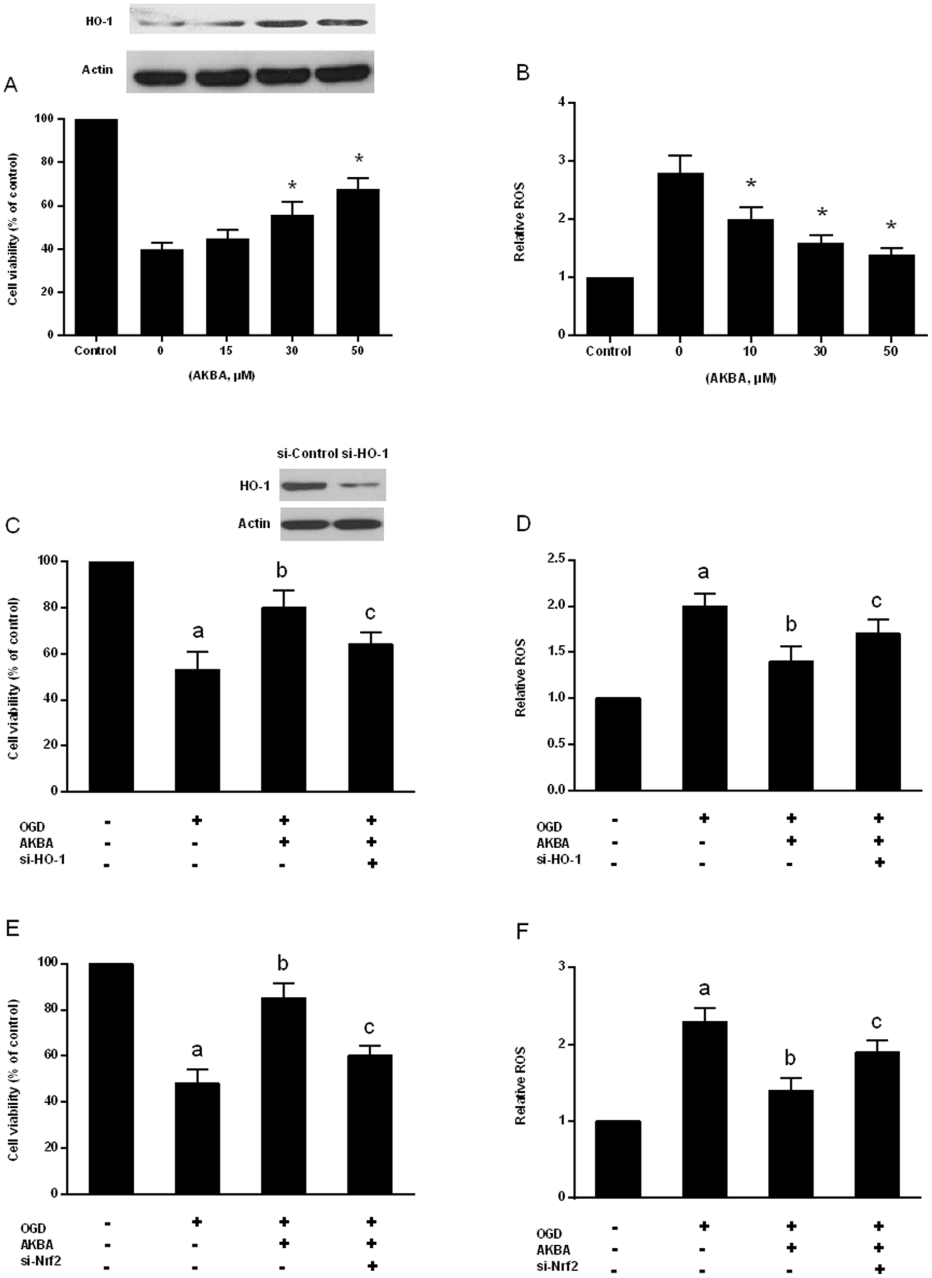
AKBA protects primary cultured neurons against OGD-induced cell death AKBA affords cell protection through the Nrf2/HO-1 pathway. Cells were pretreated with AKBA for 2 h before being subjected to 60 min OGD then incubated with AKBA for an additional 24 h. All data represent the mean ± SD of triplicate independent experiments. (A) Cell viability assay using MTT and HO-1 expression; (B) Intracellular ROS level. *, P < 0.05 vs. control. (C) Cells were treated for 48 h with control or HO-1 siRNA (transfection efficiency was checked by Western analysis), then subjected to 50 μM AKBA for 2 h before being subjected to 60 min OGD followed at 24 h by the MTT assay; (D) Intracellular ROS level. (E) Cells were treated for 48 h with control or Nrf2 siRNA, then subjected to 50 μM AKBA for 2 h before being subjected to 60 min OGD followed at 24 h by the MTT assay; (F) Intracellular ROS level. a, P < 0.05 vs. control; b, P < 0.05 vs. OGD, and c, P < 0.05 vs. AKBA-treated OGD group.

OGD treatment resulted in 60% cell death (P < 0.05). AKBA significantly blocked OGD induced cell death and increased HO-1 expression in dose dependently (P < 0.05; Figure 6A). Compared to the OGD group, the viability of the cells treated with 50 μM AKBA was increased by approximately 28% (P < 0.05). Assessment of intracellular ROS level showed that AKBA effectively reduced OGD-induced increase of intracellular ROS level in a dose dependent manner (P < 0.05; Figure 6B). The results of AKBA mediated neuroprotection were in accordance with our study in rats.

### The neuronal protection of AKBA involves the Nrf2/HO-1 pathway

To confirm the role of Nrf2/HO-1 pathway in AKBA mediated neuroprotection, we transfected control (si-Control), HO-1-specific (si-HO-1) or Nrf2-specific (si-Nrf2) siRNA in primary cultured neurons for 48 h. The efficiency of the HO-1 siRNA in knocking down HO-1 was measured by Western blot (Fig. 6C). Subsequently, the cells were treated with 50 μM AKBA, then were subjected to 1 h OGD challenge followed by reoxygenation for 24 h. Cell viability was analyzed and ROS was detected. Knockdown of HO-1 or Nrf2 inhibited cell viability that was increased by AKBA under OGD (Fig. 6C and D). In consistent, ROS level was increased by knockdown of HO-1 or Nrf2 (Fig. 6E and F). Taken together, AKBA effectively prevented neurons from oxidative damage involved activation of Nrf2/HO-1 pathway.

## Discussion

The ischemic cascade begins with a drastic disruption of cerebral blood flow that deprives brain cells of oxygen and glucose supply, leading to a decrease in energy production associated with building up toxic substances such as glutamate, inflammatory mediators, and free radicals that could ultimately result in neurodegeneration. Oxidative damage plays a cardinal role during ischemia. The protective role of Nrf2 has been highlighted by up-regulating a battery of ARE-driven antioxidative and cytoprotective genes to defend against oxidative stress^18^. Consequently, drugs that interfere with the Nrf2 pathway may have a potential as neuroprotective agents.

There is considerable current interest in the neuroprotective effects of natural antioxidants against oxidative stress. Different defense mechanisms involved can activate Nrf2 and increase expression of antioxidative genes. Due to its antioxidant properties, AKBA also has potential implications in treating oxidative injuries^4^; but, the mechanism of AKBA protection was poorly understood. Our study demonstrates for the first time that AKBA has protective effects against cerebral I/R injury in MCAO model attested by improved neurologic scores, reduced infarct volume and ameliorated neuronal apoptosis. The mechanism is possibly attributed to activating Nrf2/HO-1 pathway. Recently, triterpenoids structurally similar to AKBA, such as maslinic acid and oleanolic acid, have been reported to significantly increase Nrf2, leading to neuroprotection^7^,^19^. In another approach, stimulating Nrf2 by triterpenoid could effectively reduce 1-methyl-4-phenyl-1,2,3,6-tetrahydropyridine (MPTP)-induced oxidative stress in the mouse model while Nrf2 knockout mice failed to block against MPTP neurotoxicity, suggesting a direct protective role of Nrf2/ARE against MPTP neurotoxicity^20^. These data are noteworthy and suggest that specific triterpenoid compounds could be beneficial for the treatment of ischemic stroke associated with oxidative stress.

We discovered that AKBA, as a novel Nrf2 activator, enhanced the protective defense mechanisms through the HO-1 neuroprotective pathway. In this study, AKBA markedly increased the expression of nuclear Nrf2 and total HO-1 in ischemic cortex at 48 h after MCAO as well as in primary culture of rat cortical neurons. In support of this notion, the double immunofluorescent studies revealed that in cortex the neuronal HO-1 was clearly upregulated. On the subcellular localization of Nrf2, more nuclear immunofluorescence was profoundly visualized in neurons at the cortex than sham controls, indicating nuclear translocation of Nrf2 in neurons under the oxidative stress after MCAO. On the other hand, consistent with our findings, Nrf2 and HO-1 induction have been reported previously in the rat brain following transient focal ischemia^21^,^22^. Expression of HO-1 in neurons is usually detected in response to stimuli such as ischemia or oxidative stress^23^.

Nrf2 is a key regulator of endogenous antioxidant defense. Under physiological situations, Nrf2 is bound by its cytosolic inhibitor, Kelch-like ECH-associated protein 1 (Keap1), and resides in the cytoplasm before it is targeted for proteosomal degradation^24^. Activation and nuclear accumulation of Nrf2 upregulates endogenous antioxidant defences to restore cellular redox homeostasis via the induction of phase II defence enzymes. Existing data has demonstrated that Nrf2-deficient mice are more susceptible to oxidative stress^25^. Another study on wild type and Nrf2 knockout mice also showed that Nrf2 reduces ischemic brain injury by protecting against oxidative stress^26^. The HO-1 promoter is known to have a large number of ARE sequences to which Nrf2 can bind to induce its expression in a preferential manner^27^. As shown in our *in vitro* study, the si-Nrf2 treatment significantly decreased the level of Nrf2 in nuclear extracts from cells treated with AKBA, and reduced the up-regulation of its target gene HO-1.

Noteworthy, the expression of HO-1 is mediated by several signaling pathways and transcription factors, including Nrf2, AP-1, and NF-kB as the most prominent^28^. Among them redox-dependent Keap1/Nrf2 system plays a central role for HO-1 induction in response to oxidative stress. HO-1 has been implicated to be particularly important in neuroprotection against cerebral ischemia, as evidenced by HO-1 knockout mice exhibiting greater ischemic damage as compared to wild type mice^29^.

SOD dismutates superoxide to hydrogen peroxide and oxygen^30^. MDA is not only produced by oxidative stress-induced peroxidation, but additionally by enzymatically produced lipid peroxidation during the arachidonic acid cascade which is an important element of post-ischemic secondary injury^30^. The decreased activities of SOD and enhanced MDA level in the vehicle group imply that severe oxidative stress occurred during permanent MCAO that increased free radical activity and reciprocally reduced endogenous antioxidants occurring during cerebral ischemia. Our results are consistent with previous work^4^,^9^. With AKBA treatment, enhanced activity of SOD and a decreased level of MDA were found. We propose that an in vivo therapeutic effect of AKBA is related to an antioxidant effect by enhancing Nrf2 regulation and therefore alleviates oxidative stress during ischemic stroke.

As stated by the MTT assay and ROS measurement *in vitro*, pretreatment with AKBA before OGD damage can significantly reduce cell death. We speculate that the protective effect was due to the antioxidant properties of AKBA. Increased levels of ROS are the major cause of tissue injury after cerebral ischemia, in which inactivation of antioxidant enzymes and consumption of antioxidants generated by endogenous antioxidant defense mechanisms fail to protect neurons from oxidative damage^31^. In neuronal viability assays, AKBA reduced neuronal cell death triggered by OGD. The fact that AKBA could inhibit the increase in ROS also shows that AKBA may have the ability to rescue cells from OGD. These *in vitro* data support our in vivo results and show that AKBA promotes an antioxidative effect.

Another key finding of the current study was that knockdown of HO-1 in primary cultured cortical neurons that were subjected to OGD partly diminished neuroprotective effect of AKBA. These observations strongly indicate that Nrf2/HO-1 is required for AKBA-dependent cytoprotection against oxidative stress, although other enzymes can also assist with AKBA neuroprotection. ARE is a nucleotide sequence located on the enhancer region of genes that encode phase II detoxifying enzymes, such as NQO1, HO-1, and GCLC^32^. When cells are subjected to oxidative or xenobiotic stress, Nrf2 activates the expression of several dozen such cytoprotective genes by binding to the ARE^33^. Our *in vivo* studies demonstrated that AKBA increased the levels of the Nrf2 and enhanced its binding to the ARE, therefore stimulating the transcriptional activity of Nrf2. AKBA induced HO-1 expression by stimulating Nrf2-ARE binding activity. Although the mechanisms leading to nuclear translocation of Nrf2 are poorly defined, we believe that Nrf2 and HO-1 induced by AKBA decreased ROS in cortical neurons, and that it is responsible, at least in part, for the protective effects against OGD.

AKBA in preventing focal cerebral ischaemic damage in addition to the reality that AKBA is an all-natural plant-derived compound's potency makes AKBA a promising therapeutic agent. Previous clinical trials indicated that boswellic acids had lower toxicity and were well tolerated in humans^34^. However, several issues still must be addressed in the future, including whether other signaling pathways contribute to the neuroprotective effects of AKBA. It should be mentioned that AKBA is a 5-lipoxygenase inhibitor and its own anti-inflammatory effect *in vivo* will likely contribute to the protective effects of this compound in stroke^35^. For the short-term study (48 h), we confirmed the protective effects of AKBA on the acute phase of stroke. Therefore, therapeutic time window of AKBA injection against cerebral I/R injury in rat should be determined in future and the possible long-term curative effects of AKBA remain to be clarified.

In this study, we firstly demonstrated that AKBA could attenuate ischemic neuronal injury using a MCAO model. Furthermore, *in vivo* studies have shown that AKBA can protect neurons against OGD-induced cell death activating the Nrf2/HO-1 pathway. The protective effect of AKBA was partly blocked when HO-1 or Nrf2 was knocked down. Collectively, we demonstrated the unexplored potential of AKBA for the treatment of cerebral I/R damage and that pharmacological activation of the Nrf2/HO-1 pathway can provide neuroprotection.

## Author Contributions

Y.D., Y.W.L. and A.D.W. conceived and designed the experiments. M.C.C., M.M.W. and Y.S. performed the experiments. Y.Y.J. and M.W. analyzed the data. M.W., T.J.Z. and J.P. contributed reagents/materials/analysis tools. Y.D., Y.R.Z. and C.G. wrote the paper. All authors reviewed and approved the final manuscript.

## Supplementary Material

Supplementary Information supplementary figures

## Figures and Tables

**Table 1:** Physiologic parameters, mean ± SD (n = 8)

	MAP (mm Hg)	pH	PaCO_2_(mm Hg)	PaO_2_(mm Hg)	Glu (mg/dl)
Preischemia					
Con	117.3 ± 7.2	7.40 ± 0.05	40.4 ± 4.0	148.6 ± 17.2	140.3 ± 8.5
I/R	120.6 ± 5.1	7.41 ± 0.04	42.6 ± 3.4	146.5 ± 13.5	138.5 ± 9.2
I/R ± AKBA	114.1 ± 6.4	7.33 ± 0.05	39.5 ± 3.7	147.5 ± 15.6	137.1 ± 8.7
10 min after reperfusion					
Con	110.8 ± 6.0	7.36 ± 0.06	39.4 ± 5.1	142.1 ± 14.4	142.1 ± 10.6
I/R	105.5 ± 4.3	7.38 ± 0.06	39.1 ± 4.7	134.0 ± 12.8	145.5 ± 8.9
I/R + AKBA	107.1 ± 5.3	7.40 ± 0.05	40.2 ± 4.5	132.7 ± 15.3	141.6 ± 8.7
90 min after reperfusion					
Con	106.8 ± 7.6	7.41 ± 0.06	38.2 ± 3.9	140.0 ± 16.4	142.9 ± 8.5
I/R	100.3 ± 4.2	7.39 ± 0.04	37.4 ± 5.1	122.5 ± 11.2	148.6 ± 6.5
I/R + AKBA	101.7 ± 7.1	7.38 ± 0.05	37.8 ± 5.0	126.6 ± 14.5	143.0 ± 7.2

MAP, Mean arterial blood pressure; PaO_2_, arterial oxygen tension; PaCO_2_, arterial carbon dioxide tension; Glu, blood glucose.
