# Author Correction: Neuroprotection by acetyl-11-keto-β-boswellic acid, in ischemic brain injury involves the Nrf2/HO-1 defense pathway

**DOI:** 10.1038/s41598-022-15106-9

**Published:** 2022-07-07

**Authors:** Yi Ding, MinChun Chen, Min Wang, MingMing Wang, Tiejun Zhang, Jongsun Park, YanRong Zhu, Chao Guo, YanYan Jia, YuWen Li, AiDong Wen

**Affiliations:** 1 Department of Pharmacy, Xijing Hospital, Fourth Military Medical University, Xi’an, China; 2 Department of Pharmacology, Chungnam National University, Daejeon, South Korea; 3 Department of Pharmacology, School of Pharmacy, Fourth Military Medical University, Xi’an, China

Correction to: *Scientific Reports* 10.1038/srep07002, published online 11 November 2014

This Article contains errors.

In Figure 5A, the image for His H3 was inadvertently duplicated from Figure 5A. A corrected version of Figure 5 and its accompanying legend are included below.

**Figure 5:**
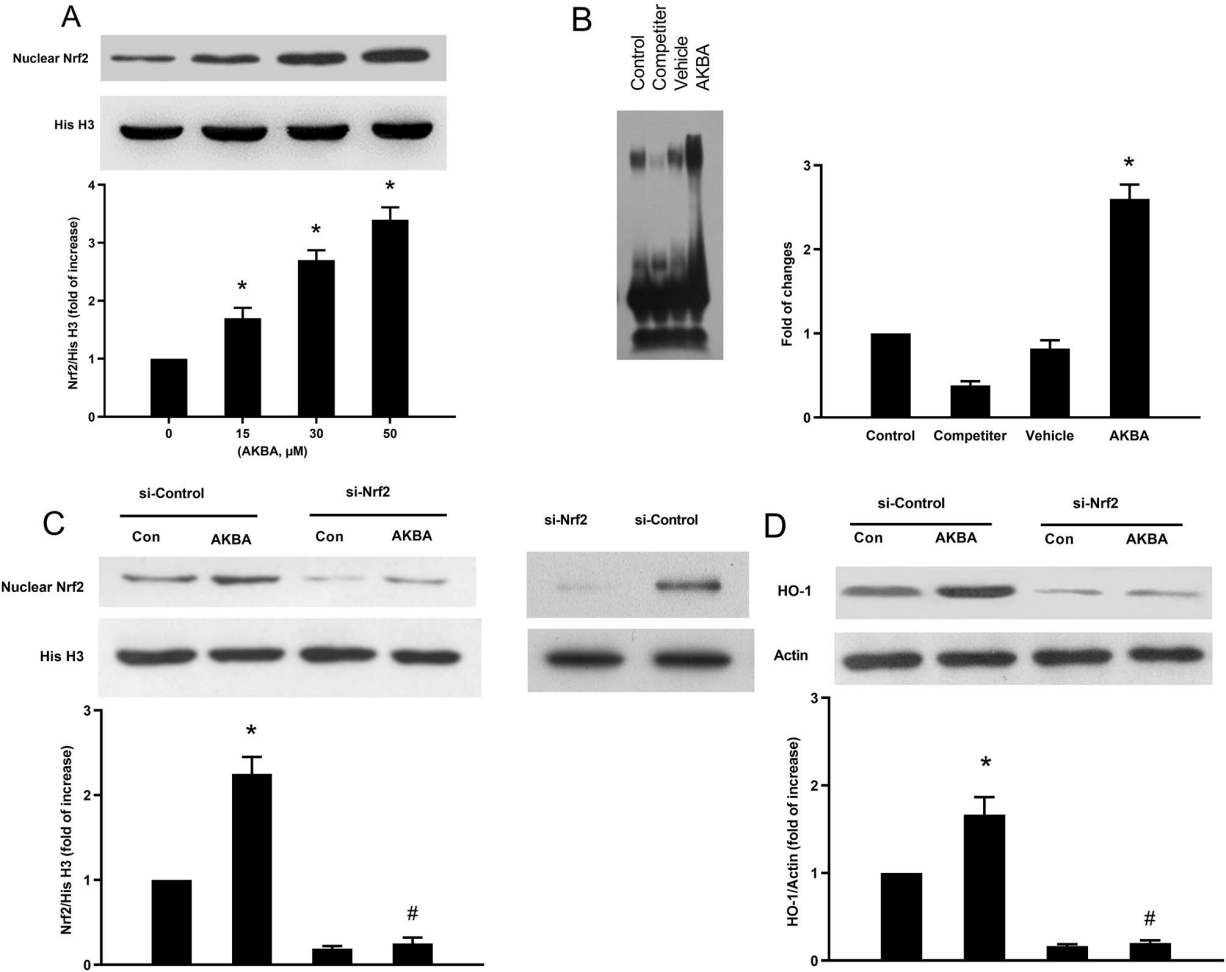
AKBA induces expression of Nrf2 and Nrf2-binding activity in primary cultured neurons. All data represent the mean ± SD of triplicate independent experiments. (**A**) AKBA induced Nrf2 expression in a concentration-dependent manner. *P < 0.05 vs control (**B**) After 2 hour treatment with vehicle or 50 μM AKBA, nuclear extracts were prepared and were used to analyze Nrf2 bingding activity by EMSA. (**C**) Cells were transiently transfected with control or Nrf2 siRNA for 48 h (transfection efficiency was checked by Western analysis), followed by treatment with 50 μM of AKBA for an additional 8 h. Nuclear extracts were analyzed for Nrf2 levels. (**D**) Representative immunoblots for HO-1 following 50 μM of AKBA treatment for 24 h in control and Nrf2 siRNA-treated cells. *P < 0.05 vs si-control group without AKBA and ^#^P < 0.05 vs si-control group with AKBA.

