# Supplementary material for: Neuroprotection by Acetyl-11-Keto-β-Boswellic Acid, in Ischemic Brain Injury Involves the Nrf2/HO-1 defense Pathway
Source: Sci Rep. 2014 Nov 11;4:7002. doi: 10.1038/srep07002 (PMC4227012; doi:10.1038/srep07002)

Supplementary data

**Neuroprotection by Acetyl-11-Keto-β-Boswellic Acid, in** **Ischemic Brain Injury Involves the Nrf2/HO-1 defense Pathway**

Running title: Neuroprotection of AKBA by Nrf2/HO-1

Authors: Yi Ding^1a^; MinChun Chen^1a^; Min Wang^3a^; MingMing Wang^1^^a^; Tiejun Zhang^2^; Jongsun Park^2^; YanRong Zhu^1^; Chao Guo^1^; YanYan Jia^1^; YuWen Li^1^*****; **AiDong Wen**^1^*****

a: These authors contributed equally to this work.

*: Corresponding Authors: YuWen Li (liyuwenzs@gmail.com) and **AiDong Wen**

**(adwen-2004@hotmail.com)**

1: Department of Pharmacy, Xijing Hospital, Fourth Military Medical University

2: Department of Pharmacology, Chungnam National University

3: Department of Pharmacology, School of Pharmacy, Fourth Military Medical University

Figs 1. With the treatment of AKBA, infarct volume was greatly reduced in a dose-dependent manner as compared with the vehicle group. *P<0.05 AKBA versus vehicle treated group. # P<0.05. 20 mg/kg AKBA group versus 10 mg/kg AKBA treated group. (n=6 animals for each group)


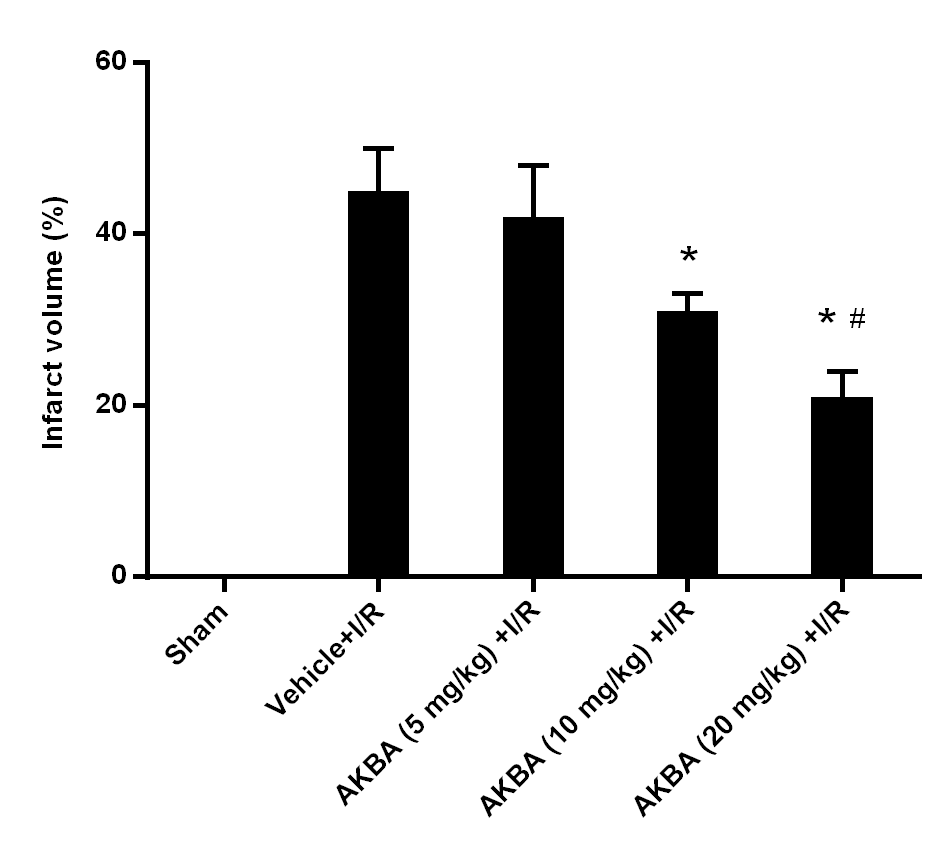


Figs 2. There is no significant difference in SOD activity (A), MDA levels (B) and HO-1 expression (C) in brain tissue, between Sham group and Sham+AKBA group. Data are presented as mean±SD. (n=6 animals for each group)


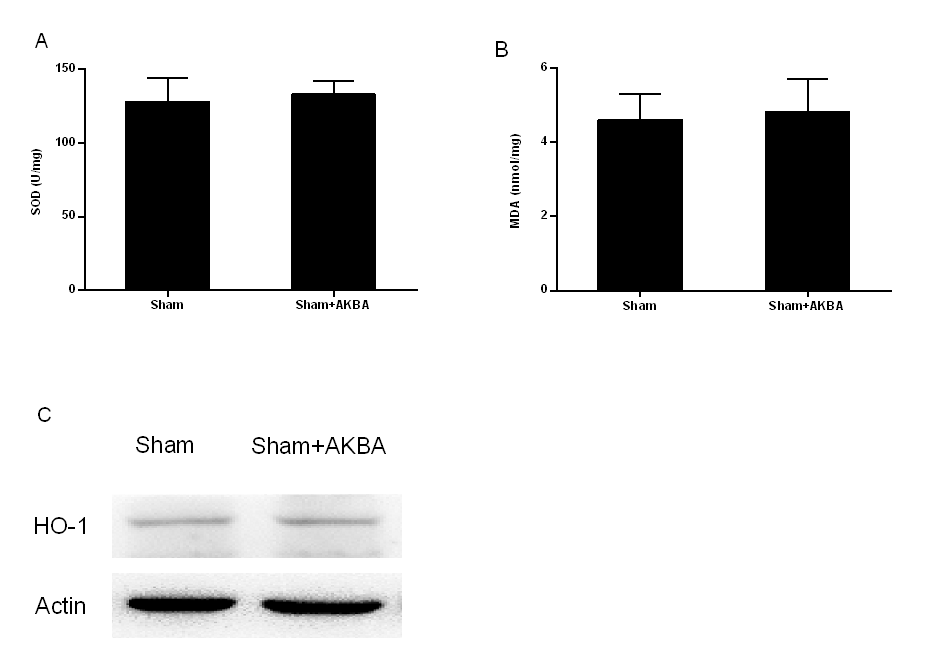

Supplement: Supplementary Information — supplementary figures [file srep07002-s1.docx]
